# Muscle Biomarkers in Colorectal Cancer Outpatients: Agreement Between Computed Tomography, Bioelectrical Impedance Analysis, and Nutritional Ultrasound

**DOI:** 10.3390/nu16244312

**Published:** 2024-12-13

**Authors:** Andrés Jiménez-Sánchez, María Elisa Soriano-Redondo, María del Carmen Roque-Cuéllar, Silvia García-Rey, Manuel Valladares-Ayerbes, José Luis Pereira-Cunill, Pedro Pablo García-Luna

**Affiliations:** 1 Unidad de Gestión Clínica de Endocrinología y Nutrición, Instituto de Biomedicina de Sevilla, IBiS/Hospital Universitario Virgen del Rocío/CSIC/Universidad de Sevilla, Avda. Manuel Siurot s/n, 41013 Seville, Spain; mariac.roque@juntadeandalucia.es (M.d.C.R.-C.); silviam.garcia@juntadeandalucia.es (S.G.-R.); jpereira2@us.es (J.L.P.-C.);; 2 Unidad de Gestión Clínica de Radiodiagnóstico, Hospital Universitario Virgen del Rocío, Avda. Manuel Siurot s/n, 41013 Seville, Spain; 3 Unidad de Gestión Clínica de Oncología Médica, Instituto de Biomedicina de Sevilla, IBiS/Hospital Universitario Virgen del Rocío/CSIC/Universidad de Sevilla, Avda. Manuel Siurot s/n, 41013 Seville, Spain; mvalaye@icloud.com

**Keywords:** muscle mass, validation study, computed tomography, bioelectrical impedance analysis, nutritional ultrasound^®^, obesity, GLIM, sarcopenia, colorectal cancer

## Abstract

Background: Muscle quality and mass in cancer patients have prognostic and diagnostic importance. Objectives: The objectives are to analyze agreement between gold-standard and bedside techniques for morphofunctional assessment. Methods: This cross-sectional study included 156 consecutive colorectal cancer outpatients that underwent computed tomography (CT) scanning at lumbar level 3 (L3), whole-body bioelectrical impedance analysis (BIA), point-of-care nutritional ultrasound^®^ (US), anthropometry, and handgrip strength in the same day. Measured muscle biomarkers were stratified by sex, age, BMI-defined obesity, and malnutrition using Global Leadership in Malnutrition (GLIM) criteria. Whole-body estimations for muscle mass (MM) and fat-free mass were calculated using two different equations in CT (i.e., Shen, and Mourtzakis) and four different equations for BIA (i.e., Janssen, Talluri, Kanellakis, and Kotler). Muscle cross-sectional area at L3 was estimated using the USVALID equation in US. Different cut-off points for muscle atrophy and myosteatosis were applied. Sarcopenia was defined as muscle atrophy plus dynapenia. Intra-technique and inter-technique agreement were analyzed with Pearson, Lin (*ρ*), and Cohen (*k*) coefficients, Bland–Altman analyses, and hypothesis tests for measures of central tendency. Results: Intra-technique agreements on muscular atrophy (CT *k* = 0.134, BIA *k* = −0.037, US *k* = 0.127) and myosteatosis (CT *k* = 0.122) were low, but intra-technique agreement on sarcopenia in CT was fair (*k* = 0.394). Inter-technique agreement on muscular atrophy and sarcopenia were low. Neither CT and BIA (*ρ =* 0.468 to 0.772 depending on equation), nor CT and US (*ρ =* 0.642), were interchangeable. Amongst the BIA equations, MM by Janssen proved the best, with a 1.5 (3.6) kg bias, (−5.6, 8.6) kg LoA, and 9/156 (5.7%) measurements outside the LoA. Muscle biomarkers in all techniques were worse in aged, female, or malnourished participants. Obesity was associated with higher muscle mass or surface biomarkers in all techniques. Conclusions: Bedside techniques adequately detected patterns in skeletal muscle biomarkers, but lacked agreement with a reference technique in the study sample using the current methodology.

## 1. Introduction

Computed tomography is a gold-standard body composition technique [1]. Software-based skeletal muscle segmentation at lumbar level 3 allows for the measurement of skeletal muscle area (L3-SMA) and density (L3-SMD). SMA can be used directly, indexed by height to produce the Skeletal Muscle Index (SMI), or used in different regression equations to obtain whole-body muscle mass (MM) [2] or fat-free mass (FFM) [3] estimates to diagnose low muscle mass (muscle atrophy). Muscle atrophy is part of the Global Leadership Initiative on Malnutrition (GLIM) [4] diagnostic criteria for malnutrition. As sarcopenia is a combination of muscle atrophy plus low strength (dynapenia), muscle atrophy is also part of the European Working Group on Sarcopenia in Older People (EWGSOP) diagnostic criteria [5]. In colorectal cancer patients, CT-determined muscle atrophy works as an independent predictor of survival and postoperative complications [6], functionality [7,8], and quality of life [9], amongst others. SMD acts as a surrogate measure for fatty infiltration (myosteatosis), a condition that associates worse prognosis and functionality in colorectal cancer [8,10]. Although it is an ionizing technique with limited accessibility, CT allows for opportunistic or retrospective measurements in routine studies requested in medical or surgical services for diagnostic–therapeutic purposes.

Bedside body composition analysis techniques, such as bioelectrical impedance analysis (BIA) and nutritional ultrasound^®^ (US), are innocuous, relatively fast, portable, and cheaper. BIA is a doubly indirect technique that measures the following pure or raw bioelectrical parameters: Z (impedance), R_z_ (resistance), X_c_ (capacitive reactance), and phase angle (θ). These parameters may be introduced alongside age, sex, and basic anthropometric data in regression equations that estimate MM or FFM as measured by a reference technique [11,12]. US is an indirect technique that can evaluate muscular and adipose structures both in quantitative (thickness and area) and qualitative (echogenicity) terms. Using the nutritional ultrasound^®^ methodology, muscle measurements take place in the distal third of the imaginary segment that joins the anterior superior iliac spine and the upper border of the patella, evaluating both the rectus femoris and vastus intermedius muscles [13]. US remains stable in fluid overload situations [14,15] and may predict muscle functionality [16]. Although it can diagnose myosteatosis based on echo-intensity levels, measurements from different devices may not coincide [17].

As previously stated, “The global clinical nutrition community needs to work together to come to consensus on the optimal tool(s) to use to assess nutrition status at the bedside” [18], and further studies that analyze agreement between CT and bedside body composition techniques are still needed [19]. To our knowledge, a simultaneous cross-sectional evaluation of the quantitative and qualitative agreement between a reference technique and two bedside techniques (BIA and US) in a colorectal cancer population has not yet been carried out. Performing all techniques on the same day avoids changes in body composition due to treatment or clinical evolution in an inter-technique time interval. If these techniques were directly interchangeable under the currently state of methodology and procedures, this could allow for more diagnostic efficiency and better continuity of care: US and BIA could be performed in the scheduled follow-up by Nutrition units, while CT scans requested for diagnostic or staging reasons could be analyzed opportunistically in between, thus having a closer follow-up of the subject’s muscle status. Since CT scans can be analyzed retrospectively and with telemedicine, this closer monitoring would go unnoticed and without inconvenience for the patient. The aim of the present study is to analyze the degree of agreement in a simultaneous body composition analysis using CT, BIA, US, and anthropometry in a real-life sample of outpatients under follow-up for colorectal cancer, regardless of their treatment modality.

Our research questions were the following:

- Can all techniques detect similar patterns in body composition in the study sample?
- Are a reference (CT) and bedside techniques (BIA, US) interchangeable using current regression equations for MM or FFM in the study sample?
- How do current operational definitions of muscle atrophy, myosteatosis, and sarcopenia agree in the study sample?

Our hypothesis was that all techniques may detect similar patterns in the study sample, but direct interchangeability may not exist neither intra-technique nor inter-technique.

## 2. Materials and Methods

### 2.1. Study Design

This cross-sectional validation study was carried out in a single center (Hospital Universitario Virgen del Rocío, Seville, Spain). Consecutive sampling was used, and measurements took place from July 2022 to June 2023. This study protocol was designed following STROBE [19] and QUADAS-2 [20] recommendations.

The inclusion criteria were:

- Colorectal cancer outpatients over 50 years of age;
- Under active surveillance by the Oncology Department;
- With a programmed abdominal CT scan;
- Eastern Cooperative Oncology Group (ECOG) performance status of 0 to 3.

The exclusion criteria were

- ECOG 4-5;
- Terminal illness;
- Presence of a pacemaker, implantable cardioverter defibrillator, or intrathecal pain pump;
- Skin lesions or severe adhesive dermatitis that contraindicated electrode placement in the required areas for BIA;
- Severe cognitive impairment;
- Medical conditions that may artifact measurements at the discretion of the researcher (stroke with right residual hemiplegia, amyotrophic lateral sclerosis, or muscular dystrophy that may affect BIA and US; symptomatic rheumatoid arthritis or gouty arthritis of hand and wrist that may affect HGS);
- Unable to perform an informed consent or no desire for participation.

All measurements took place in the same day and order in a room with centrally-controlled temperature and devoid of strong electromagnetic fields. Liquid and solid intake was prohibited in the last two and eight hours, respectively. No patients received enteral or intravenous fluids during the fasting or measurement period, and no diuretic treatment was allowed during the fasting period. After emptying the bladder in a contiguous WC, participants undressed. Their clothing, smartphone, wearables, and jewelry were stored away. After a brief physical exploration looking for possible artifacts (central catheter, edema, or ostomy), participants rested in a supine position in an electrically isolated stretcher, covered by a hospital blanket. US was performed, with BIA measurements taking place immediately afterwards. Next, height, weight, and abdominal circumference were measured and annotated. Then, participants got dressed and handgrip strength was measured with a handheld dynamometer. Finally, patients were sent to Radiology so they could undergo their corresponding abdominal CT scan following normal procedures.

This study was conducted in accordance with the Declaration of Helsinki and approved by the Ethics Committee “CEI de los Hospitales Universitarios Virgen Macarena y Virgen del Rocío” (protocol code: 1006-N-22; date of approval: 23 May 2022). All patients participating in this study gave informed consent. “Written informed consent was conducted in person by A.J.-S. Each participant was provided with a written document detailing why they were contacted, what the participation consisted of, its possible risks and benefits, and pseudonymization of data. Only A.J-S. accessed the two separate password-encrypted databases of this study (one for patient identification, and one for data), which have been kept in custody in accordance with current European and Spanish legislation”.

### 2.2. Procedures

#### 2.2.1. Computed Tomography (CT) Scan Segmentation

Abdominal CT scans were requested by the Oncology Department of our center due to diagnostic–therapeutic reasons. Both the General Electric Revolution EVO (GE HealthCare Technologies Inc., Chicago, IL, USA) and Toshiba Aquilion (Toshiba, Minato, Japan) scanners were used. Portovenous phase scans with a slice thickness of either 1.00 or 1.25 mm were obtained after intravenous administration of a contrast medium following a standardized acquisition protocol. The images were retrospectively downloaded in Digital Imaging and Communication in Medicine (DICOM). DICOM files were then anonymized using DICOM Anonymizer v2.4.2 (https://www.dicomanonymizer.com/index.html, accessed on 1 November 2024).

Tissue segmentation was performed with Horos (Annapolis, MD, USA). Its 4.0.0RC4 version was used for this study (https://github.com/horosproject/horos/releases, accessed on 1 November 2024). The rationale behind its use has been previously explained [21].

All segmentations were performed on a Mac Mini M1 with 16 GB of RAM (Apple Inc., Cupertino, CA, USA) and an LG 32UN500P-W 31.5-inch screen with 4K resolution (LG Electronics, Seoul, Republic of Korea). The identification of the L3 vertebra and skeletal muscle segmentation using a −29 to +150 Hounsfield units (HU) threshold in a selected axial slice were performed following the Alberta protocol (TomoVision, Magog, QC, Canada, https://tomovision.com/Sarcopenia_Help/index.htm, accessed on 1 November 2024). Both the skeletal muscle cross-sectional area (L3-SMA) in square centimeters (cm^2^) and skeletal muscle density (L3-SMD) in Hounsfield units (HU) were recorded.

#### 2.2.2. Bioelectrical Impedance Analysis (BIA)

Bioelectrical impedance was measured with a phase-sensitive touch screen impedance device (Nutrilab™, Akern SRL; Pontassieve, Florence, Italy), working with an alternating sinusoidal electric current of 230 μA at an operating frequency of 50 kHz (±1%). The device was calibrated every morning using the standard control circuit supplied by the manufacturer with a known impedance resistance (R_z_) = 380 Ω; reactance (X_c_) = 45 Ω. Impedance data are shown directly in a LCD touchscreen and stored into an internal memory. Accuracy: R_z_: ±0.1 Ω; X_c_: ±0.1 Ω; CV% <1%. The pure bioelectric parameters obtained were: Z (in Ω), R_z_ (in Ω), X_c_ (in Ω) and θ (θ = tan^−1^ (X_c_/R_z_), in °). The measurement technique was conducted according to ASPEN and ESPEN recommendations [18,22], with arms separated at 30° and legs at 45°. The electrode placement area was cleaned with 70° alcohol. Once dry, two sets of adhesive Ag/AgCl low impedance electrode (Bivatrodes™, Akern Srl; Pontassieve, Florence, Italy), designed for accurate and sensitive bioimpedance measurements, were placed proximal to the phalangeal–metacarpal joint on the dorsal surface of the right hand and distal to the transverse arch on the superior surface of the right foot. Sensor electrodes were placed at the midpoint between the distal prominence of the radius and ulna of the right wrist, and between the medial and lateral malleoli of the right ankle. The clamps of the measuring cable were then attached, avoiding the occurrence of loops. After an approximate time of five minutes in the supine position, participants underwent three consecutive measurements and each R, X_c_, and θ were registered. Its respective means were used as input in Bodygram HBO version 3.1.6 (Akern SRL, Pontassieve, Florence, Italy) to calculate Talluri equations for both MM and FFM, or RStudio version 2023.06.1 + 524 to calculate other MM and FFM equations (Janssen, Kanellakis, and Kotler).

#### 2.2.3. Nutritional Ultrasound^®^ (US)

US was performed using a Butterly iQ+^TM^ (Butterfly Network Inc., Burlington, MA, USA), a point-of-care (POC) handheld device with a 1–10 MHz range and a built-in battery that was connected via cable to a Samsung Galaxy S9 smartphone (Samsung Group, Suwon, Republic of Korea). Images were recorded using the “Musculoskeletal/Soft Tissue” presets, and the depth was adjusted as needed to visualize all structures of interest following previous recommendations [13]. All recorded images were stored in the Butterfly Network in a pseudo-anonymized manner. Images where then downloaded as .PNG files and measured in ImageJ in the same computer where CT scan segmentation took place. After calibrating each image measuring two centimeters in its built-in scale with the “Straight” tool and the Shift command to ensure a straight line, the “Set Scale” tool was then used to set the scale for that given image. Both Quad-MT (the whole depth of the vastus intermedius plus the rectus femoris of the quadriceps muscle in millimeters) and RF-MT (the depth of the rectus femoris of the quadriceps muscle in millimeters) were measured using the “Straight” tool. RF-CSA (the rectus femoris cross-sectional area in square centimeters) was measured using a manual “Polygon” tool.

#### 2.2.4. Anthropometry Protocol

Height and weight measurements were performed according to ASPEN recommendations [18]. Height was measured with a SECA wall-mounted measuring rod (seca GmbH & Co. KG, Hamburg, Germany), installed at 200 mm. Weight was measured using a new and calibrated portable weight CAS-PB-150 (CAS Corporation, Seoul, Republic of Korea), with a capacity of up to 150 kg and two ranges of measuring sensitivity (20-g differences up to 60 kg; 50-g differences between 60 and 150 kg).

#### 2.2.5. Handgrip Strength (HGS) Measurements

Handgrip strength was measured in kilograms (kg) using a JAMAR Plus handheld dynamometer (Performance Health Sammons Preston, Warrenville, IL, USA) in the dominant side following the Southampton protocol [23], and the best of three attempts was used for this study. Further details are discussed elsewhere [24].

#### 2.2.6. Clinical Variables and Cancer Staging

The following clinical variables were obtained from digitized health records (“DIRAYA Clinical Station”): date of birth and age, ECOG Performance Status [25], type of baseline disease, tumor staging (according to AJCC-TNM 8th edition) [26], type and date of treatment, previous weight, active treatment, and presence of metallic artifacts. Different systemic anticancer therapies were applied following standard schemes according to Oncology guidelines [27].

#### 2.2.7. Operative Definitions of Muscle Atrophy, Myosteatosis, Dynapenia, Sarcopenia, Malnutrition, and Obesity

Muscle atrophy in CT and BIA was defined on multiple muscle biomarkers using different cut-off points, applying EWGSOP-II [5] and GLIM [4] recommendations whenever possible (Table 1). Myosteatosis was defined by applying different cut-off points on SMD (Table 1). BIA equations were compared with CT biomarkers, including whole-body prediction equations for MM (Shen et al. [2]) and FFM (Mourtzakis et al. [3]). In US, muscle atrophy was based on the “confirmed sarcopenia” cut-off points from the DRECO study in Spanish inpatients with a simultaneous US, BIA and physical performance evaluation [28].

Dynapenia was defined as a maximal handgrip strength below the age-adjusted 10th percentile of normative values developed by Dodds et al. [40], and sarcopenia was defined as the conjunction of muscle atrophy and dynapenia using the EWGSOP-II criteria [5]. Malnutrition was defined using GLIM criteria [41]. Participants were considered to meet the etiological criterion for inflammation. Only involuntary weight loss was taken into consideration, as some participants were under a lifestyle modification to voluntarily reduce weight. Involuntary weight loss was defined as either moderate (>5% within the last 6 months or >10% beyond 6 months) or severe (>10% within the last 6 months or >20% beyond 6 months). Body Mass Index (BMI)-based malnutrition was considered as follows: “moderate” in participants with BMI < 20.0 kg/m^2^ if <70 years old or BMI < 22.0 kg/m^2^ if >70 years old, and “severe” in participants with BMI < 18.5 kg/m^2^ if <70 years old or BMI < 20.0 kg/m^2^ if >70 years old. Obesity was defined as BMI ≥ 20.0 kg/m^2^.

### 2.3. Data Quality

All techniques (CT segmentation, BIA, US, HGS, and anthropometry) were performed and analyzed by a single researcher with previous experience in body composition analysis (A.J.S.). All participants received both the gold standard (CT) and index techniques (BIA, US, HGS, and anthropometry) in the same day, and in the same order (as previously described). Image analysis was supervised by a certified radiologist (ME.S.-R.) with extensive experience in abdomen imaging. Cancer stagings, treatments, and performance scores were registered in the database as recorded by oncologists (M.V.-A.) in health records. No artificial intelligence-assisted technology was used in data analysis or manuscript preparation.

### 2.4. Statistical Analysis

The packages *tidyverse* [42], *cowplot* [43], *DescTools* [44], *ggpubr* [45], and *metan* [46] were used in RStudio software (version 2023.06.1+524) [47]. Normality was analyzed with the Shapiro–Wilk test. Normally distributed variables were depicted as mean (standard deviation), and non-normally distributed variables were described as median (interquartile range). A *t*-test (in the presence of normality and homoscedasticity) or a Wilcoxon signed-rank test were used to compare measurements of central tendency in several muscle biomarkers in each technique (CT, BIA, US, and HGS) stratifying by sex (female/male), age (less or more than 65 years), obesity, and malnutrition. These tests were also used to compare measurements of central tendency between techniques (CT vs. BIA, and CT vs. US). Simple correlation between variables was calculated with the Pearson correlation coefficient (*r*). Quantitative agreement between variables was analyzed using the Bland–Altman analysis [48], depicting for each analysis its systematic bias, standard deviation of differences, a linear regression model to analyze dose-dependent bias, and Limits of Agreement (LoA). Quantitative agreement was analyzed using Lin’s Concordance Correlation Coefficient (*ρ*), considering values > 0.99 as “near perfect”, 0.95 to 0.99 as “substantial”, 0.90 to 0.95 as “moderate”, and < 0.90 as “poor” [49]. Associated 95% Confidence Intervals (95%CI) were also calculated for each *ρ*. Categorical agreements between different muscle atrophy and malnutrition definitions were analyzed with Cohen’s kappa [50]. Outliers were not censored, and all measurements were included for statistical analysis. Statistical significance was determined in all two-tailed tests as *p* < 0.05.

## 3. Results

### 3.1. Description of the Study Sample

**Figure 1:**
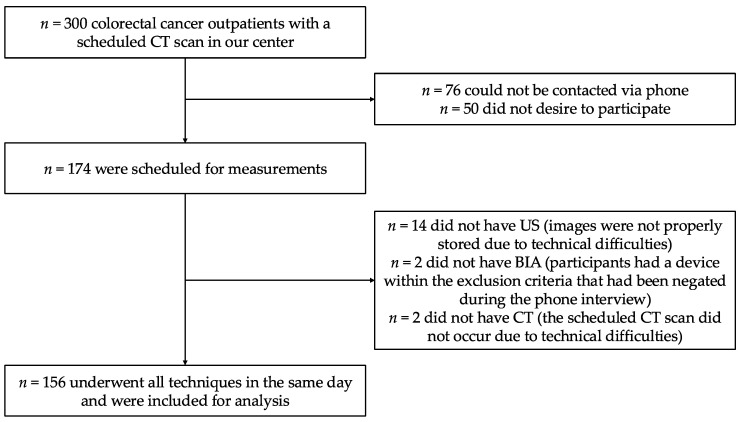
Patient recruitment, exclusions, and final sample size. BIA: bioelectrical impedance analysis; CT: computed tomography; *n* = absolute frequency; US: nutritional ultrasound^®^.

A total of n = 156 participants were measured with all techniques (CT, US, BIA, HGS, and anthropometry) and included for analysis. A flowchart summarizing patient recruitment, exclusions, and final sample size is available as Figure 1. The median time between CT and bedside techniques was 60.0 (20.0) minutes. Possible BIA artifacts (central catheter, ostomy, metallic prothesis, and diuretic) were systematically registered (Table S1), and n = 93 (59.6%) participants were completely artifact-free. No participants had fever or oedema when measurements took place.

Demographic and anthropometric data were as follows: (Table 2): *n* = 75 (48.1%) participants were female. The median age was 65.2 (13.6) years. *n* = 54 (34.6%) participants had 70 or more years. Age was 61.5 (9.8) vs. 74.6 (3.7) years (*p* < 2.2 × 10^−16^) in young vs. old participants. The median BMI was 27.3 (5.7) kg/m^2^, and the modal BMI category was overweight (36.5%)*. n* = 41 (26.3%) participants had obesity. Weight was 69.2 (17.0) vs. 90.5 (17.5) kg (*p* = 1.457 × 10^−14^) in non-obese vs. obese participants. *n* = 11 (7.0%) participants had malnutrition. All malnutrition cases were “moderate” and due to low BMI, with no significant weight loss in the study sample. Density plots for body mass index (BMI) stratified by age and sex were graphed (Figure S1).

Tumor-related characteristics were as follows (Table S1): modal ECOG was 0 (64.7%). The most frequent neoplasm was colon (41.6%), and IIIB (20.8%) was the modal TNM stage. Most (91.7%) of the participants had undergone surgery, with right hemi-colectomy (25.9%) being the most frequent surgical procedure. 93.0% of the sample received chemotherapy, mostly with adjuvant intention (62.8%). Monotherapy with capecitabine was the most frequent drug (33.8%). A total of 24.4% of the sample received chemotherapy in the 90 days before the measurements took place. Most of the participants had not received radiotherapy (69.9%).

### 3.2. Muscle Biomarkers

#### 3.2.1. General Description

**Figure 2:**
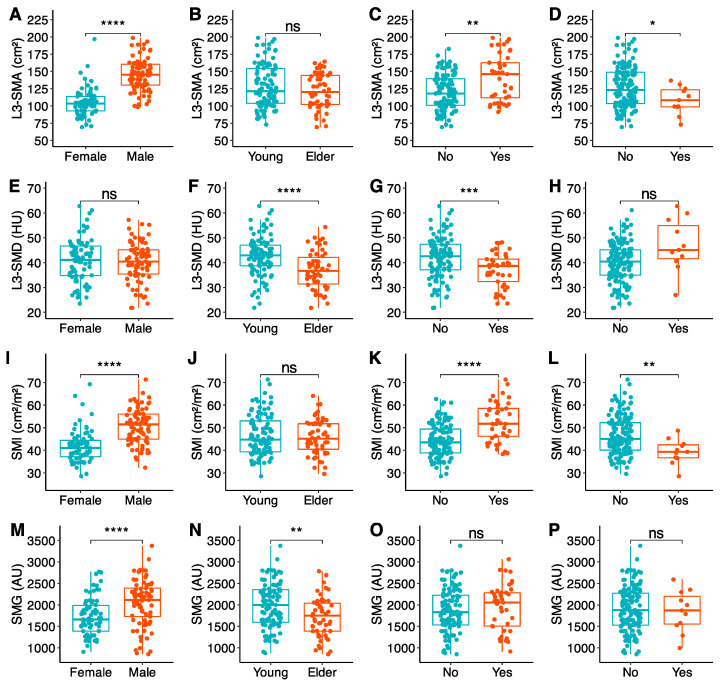
CT-based muscle biomarkers, by rows (from top to down): skeletal muscle area (**A**–**D**), skeletal muscle density (**E**–**H**), skeletal muscle index (**I**–**L**), and skeletal muscle gauge (**D**–**P**). Data are stratified in columns (from left to right) by sex (**A**,**E**,**I**), age (**B**,**F**,**J**), obesity (**C**,**G**,**K**), and malnutrition (**D**,**H**,**L**). Statistical significance for comparisons of central tendency measures is depicted as follows: *p* > 0.05 = ns; *p* ≤ 0.05 = *; *p* ≤ 0.01 = **; *p* ≤ 0.001 = ***; *p* ≤ 0.0001 = ****. AU: arbitrary uits; BMI: Body Mass Index; Cm: centimeters; GLIM: Global Leadership Initiative in Malnutrition; HU: Hounsfield units; L3-SMA: skeletal muscle area at L3; L3-SMD: skeletal muscle density at L3; m: meters; SMG: skeletal muscle gauge at L3; SMI: skeletal muscle index at L3M.

**Figure 3:**
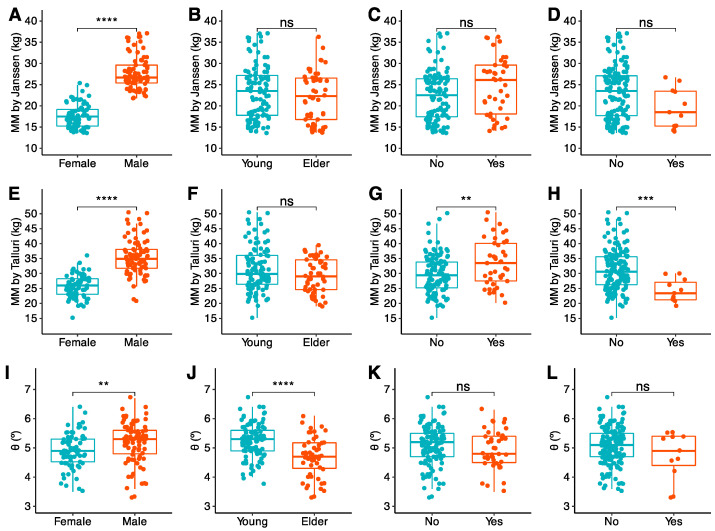
BIA-based muscle biomarkers, by rows (from top to down): Janssen et al. [34] MM (**A**–**D**), Talluri MM (**E**–**H**), and θ (**I**–**L**). Data are stratified in columns (from left to right) by sex (**A**,**E**,**I**), age (**B**,**F**,**J**), obesity (**C**,**G**,**K**), and malnutrition (**D**,**H**,**L**). °: degrees; θ: raw phase angle; MM: muscle mass. Statistical significance for comparisons of central tendency measures is depicted as follows: *p* > 0.05 = ns; *p* ≤ 0.01 = **; *p* ≤ 0.001 = ***; *p* ≤ 0.0001 = ****.

**Figure 4:**
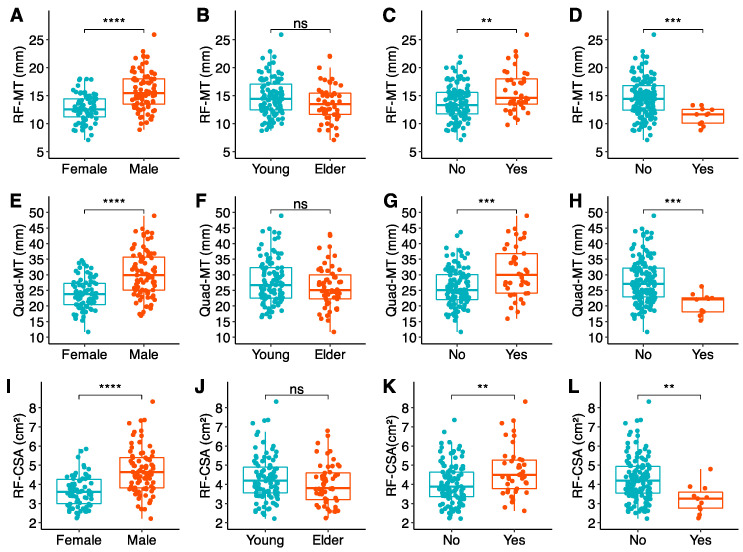
US-based muscle biomarkers, by rows (from top to down): rectus femoris muscle thickness (**A**–**D**), quadricipital muscle thickness (**E**–**H**), and quadricipital muscle area (**I**–**L**). Data are stratified in columns (from left to right) by sex (**A**,**E**,**I**), age (**B**,**F**,**J**), obesity (**C**,**G**,**K**), and malnutrition (**D**,**H**,**L**). Mm: millimeters. RF-MT: rectus femoris muscle thickness in mm; Quad-MT: quadricipital muscle thickness (rectus femoris plus vastus intermedius) in mm; RF-CSA: rectus femoris cross-sectional area in cm^2^. Statistical significance for comparisons of central tendency measures is depicted as follows: *p* > 0.05 = ns; *p* ≤ 0.01 = **; *p* ≤ 0.001 = ***; *p* ≤ 0.0001 = ****.

All analyzed muscle biomarkers in each technique (CT, BIA, US, and HGS) were stratified by sex, age, BMI, and GLIM criteria. These results and their inter-group comparisons of measures of central tendency are available in Table 3 (CT), Table 4 (BIA), and Table 5 (US and HGS). The most representative biomarkers in each technique have been graphed in Figure 2 (CT), Figure 3 (US), and Figure 4 (BIA) for further clarification and easier visualization.

#### 3.2.2. CT Muscle Biomarkers

All variables followed a non-normal distribution, except for L3-SMD and SMG. Female participants had lower muscle surface and mass biomarkers, but had no differences regarding L3-SMD. On the contrary, older participants had worse L3-SMD, but no differences regarding muscle surface and mass biomarkers. Patients with obesity had higher muscle surface and mass biomarkers (even adjusted by height), but also worse L3-SMD. Malnourished patients had worse muscle surface and mass biomarkers, and tended to have worse L3-SMD (Table 3, Figure 2).

#### 3.2.3. BIA Biomarkers

All variables followed a non-normal distribution, except for Talluri MM. Female participants had lower MM, FFM, and θ. On the contrary, older participants had worse θ, but no differences regarding MM or FFM. Patients with obesity had higher MM and FFM. They had worse θ, but without statistical significance. Malnourished patients had worse MM and FFM. They also had worse θ, but again without statistical significance (Table 4, Figure 3).

#### 3.2.4. US Biomarkers and Handgrip Strength

All variables followed a non-normal distribution. Female participants had lower US biomarkers and HGS. Older participants displayed worse HGS, but lacked differences in US biomarkers. Patients with obesity had higher US biomarkers. They had higher HGS, but without statistical significance. Malnourished patients had US biomarkers. They also had lower HGS, in this case without statistical significance (Table 5, Figure 4).

### 3.3. Muscle Biomarkers: Intra and Inter-Technique Correlation Matrices

**Figure 5:**
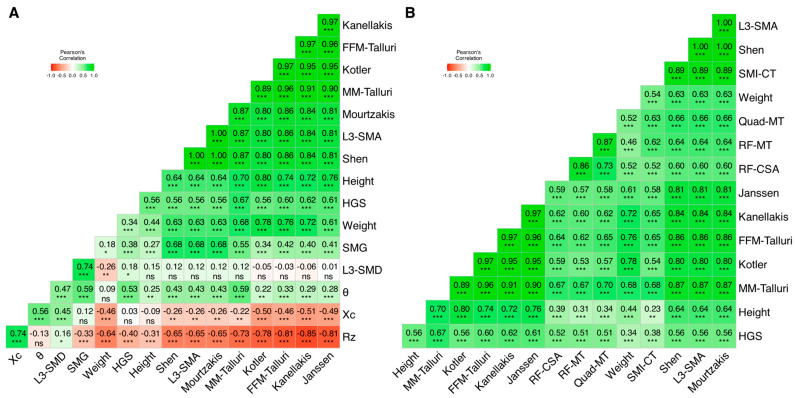
Correlation matrices, based on Spearman’s correlation coefficients (r). Direct correlations are shown in shades of green, and inverse correlations are shown in shades of red. Correlation strength is proportional to color intensity. (**A**) BIA-measured (R_z_, X_c_, and θ) parameters, BIA-estimated FFM (Kanellakis, Kotler, FFM-Talluri) [36,37] and BIA-estimated MM (Janssen, MM-Talluri) [34], CT-measured parameters (SMA, SMD), CT-estimated whole-body MM (Shen) [2] and FFM (Mourtzakis) [3], anthropometry (Height, Weight), and handgrip strength (HGS). (**B**) US-measured (Quad-MT, RF-MT, RF-CSA), BIA-estimated FFM and FM, CT-measured parameters, anthropometry, and handgrip strength. Statistical significance for comparisons of central tendency measures is depicted as follows: *p* > 0.05 = ns; *p* ≤ 0.05 = *; *p* ≤ 0.01 = **; *p* ≤ 0.001 = ***.

For an easier interpretation, muscle biomarkers were separated into two different correlation matrices. The first one includes measured and estimated parameters from BIA, measured and estimated biomarkers from CT, anthropometry, and handgrip strength (Figure 5A). The second one includes US measurements, measured and estimated biomarkers from CT, anthropometry, and handgrip strength (Figure 5B).

#### 3.3.1. Matrix 1: CT, BIA, and HGS

Regarding raw or unbiased BIA measurements:

- R_z_ had moderate inverse correlations with L3-SMA (*r* = −0.65), weight (*r* = −0.65), and HGS (*r* = −0.40), as well as a moderate direct correlation with X_c_ (*r* = 0.74).
- Conversely, X_c_ had moderate direct correlations with θ (*r* = 0.56), and L3-SMD (*r* = 0.45).
- θ had moderate direct correlations with SMG (*r* = 0.59), HGS (*r* = 0.53), L3-SMD (*r* = 0.47), and L3-SMA (*r* = 0.43).

BIA-estimated and CT-estimated whole-body muscle biomarkers were correlated as following:

- L3-SMA (in cm^2^) and all the MM equations (*r* = 0.87 Talluri; *r* = 0.81 Janssen) [34] or FFM equations (*r* = 0.86 Talluri, *r* = 0.84 Kanellakis, *r* = 0.80 Kotler) [36,37] displayed high correlation coefficients.
- Whole-body estimated MM by the Shen equation [2] and BIA-based whole-body MM equations (*r* = 0.87 Talluri; *r* = 0.81 Janssen) [34] were also strongly correlated.
- Whole-body estimated FFM by the Mourtzakis equation [3] and BIA-based whole-body FFM equations (*r* = 0.87 Talluri, *r* = 0.84 Kanellakis; *r* = 0.80 Kotler) [36,37] also showed a strong correlation.
- Maximum handgrip strength showed a moderate correlation with these parameters, being the highest for MM Talluri (*r* = 0.67).

#### 3.3.2. Matrix 2: CT, US, and HGS

All US-based muscle biomarkers had moderate direct correlations with L3-SMA, that ranged from *r* = 0.56 to *r* = 0.66, the strongest being with RF-MT (rectus femoris plus vastus intermedius). All three US-based muscle biomarkers also had a moderate or strong correlation with the other two US-based parameters, with the strongest being at RF-MT with Quad-MT (whole-quadriceps muscle thickness, *r* = 0.87) and RF-MT with RF-CSA (*r* = 0.86). The correlation with HGS was homogenous amongst all US-based biomarkers (*r* = 0.51 to *r* = 0.52).

### 3.4. Muscle Biomarkers: Agreement

#### 3.4.1. Agreement Between CT and BIA

**Figure 6:**
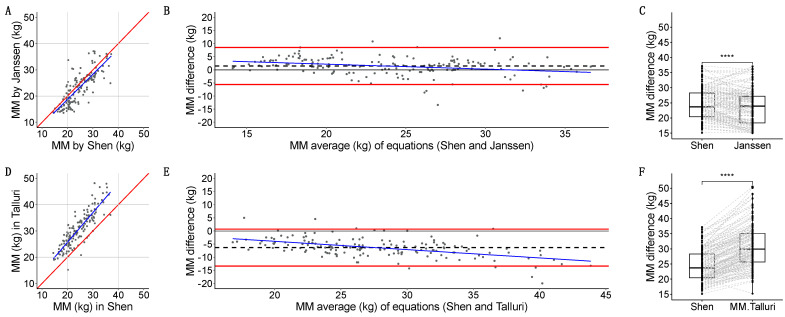
Scatterplot for CT-based MM in kg using Shen [2] equation on the *X*-axis, and different BIA-based equations for muscle mass (MM) in kilograms (kg): Janssen [34] (**A**), and Talluri (**D**) on the Y-axis. Linear regression models are represented as a blue line, and a perfect regression is represented as a red line. Bland–Altman plots for CT-based MM in kg using Shen equation, in comparison with different BIA-based MM equations: Janssen (**B**), and Talluri (**E**). In all cases, averages of MM for both equations are presented on the *X*-axis; differences in MM for both equations are presented on the Y-axis. A linear regression model to detect dose-dependent bias is represented as a blue line. Please note how the *Y*-axis has the same scale and range for easier comparisons. Individual measurements are shown as black dots; biases are represented as horizontal dashed black lines; upper and lower limits of agreement are shown as horizontal solid red lines. The absence of differences (Y intercept = 0) between equations is represented as a horizontal solid black line. Boxplots with overlaid point geometry for CT-based MM in kg using Shen equation on the *Y*-axis, and different BIA-based equations for MM in kg: Janssen (**C**), and Talluri (**F**) on the *X*-axis. Please note how the *Y*-axis has the same scale and range for easier comparisons. Each participant is joint by dashed grey lines. Statistical significances for comparisons of central tendency measures is depicted as follows: *p* ≤ 0.0001 = ****.

SMI-Janssen and SMI-CT had a moderate linear relation (r = 0.665). The agreement of different equations for whole-body MM estimation using BIA (Janssen, Talluri) [34] with the estimated whole-body MM in CT using the Shen equation [2] was analyzed:

- Janssen-estimated MM [34] had a strong linear relation with Shen [2] (*r* = 0.809, *p* < 2.2 × 10^−16^) (Figure 6A). Quantitative agreement was poor (*ρ* = 0.772, 95%CI: 0.705, 0.825). Janssen underestimated MM with a 1.5 (3.6) kg systematic bias, a minimal dose-dependent bias, (−5.6, 8.6) kg LoA, and 9/156 (5.7%) measurements outside the LoA (Figure 6B). Grouped measurements were significantly different (23.3 vs. 23.6 kg, *p* = 3.609 × 10^−8^) (Figure 6C).
- Talluri-estimated MM had a strong linear relation with Shen [2] (*r* = 0.865, *p* < 2.2 × 10^−16^) (Figure 6D). Quantitative agreement was poor (*ρ* = 0.538, 95%CI: 0.467, 0.603). Talluri overestimated MM with a −6.4 (3.6) kg systematic bias, a dose-dependent bias, (−13–41, 0.688) kg LoA, and 9/156 (5.7%) measurements outside the LoA (Figure 6E). Grouped measurements were significantly different (29.9 vs. 23.6 kg, *p* < 2.2 × 10^−16^) (Figure 6F).

As Janssen-estimated whole-body MM [34] showed the smallest bias in relation to Shen-estimated whole-body MM [2], we studied the influence of possible BIA artifacts using this equation. The Bland–Altman sub-analysis of cases that were completely artifact-free was as follows: 1.6 (3.5) kg with (−5.2, 8,4) kg LoA. Then, we visually inspected the data, looking for bias due to possible artifacts such as central catheters side (Figure S2A), and type (Figure S2B), presence of an ostomy (ileostomy, colostomy, nephrostomy) (Figure S2C), or chronic treatment with a diuretic drug (Figure S2D) in Bland–Altman plots. No systematic bias was found.

**Figure 7:**
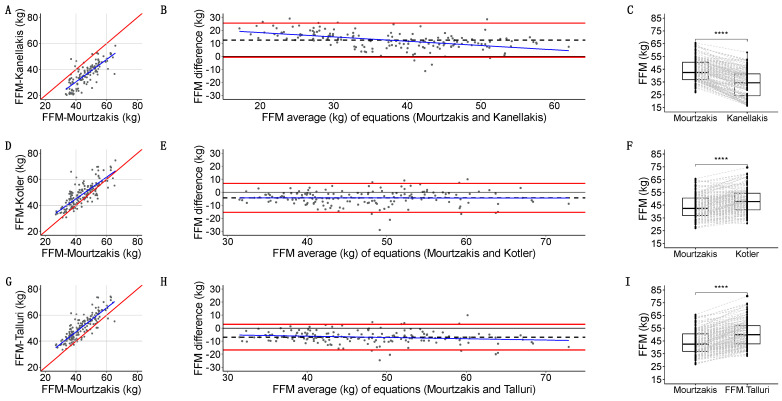
Scatterplot for CT-based FFM in kg using Mourtzakis [3] equation on the *X*-axis, and different BIA-based equations for fat-free mass (FFM) in kilograms (kg): Kanellakis [36] (**A**), Kotler [37] (**D**), and Talluri (**G**) on the *Y*-axis. Bland–Altman plots for CT-based FFM in kg using Mourtzakis equation, in comparison with different BIA-based FFM equations: Kanellakis (**B**), Kotler (**E**), and Talluri (**H**). Boxplots with overlaid point geometry for CT-based FFM in kg using Mourtzakis equation on the *Y* axis, and different BIA-based equations for FFM in kg: Kanellakis (**C**), Kotler (**F**), and Talluri (**I**) on the *X*-axis. Statistical significances for comparisons of central tendency measures is depicted as follows: *p* ≤ 0.0001 = ****.

Then, we compared the agreement of different equations for whole-body FFM estimation using BIA (Kanellakis, Kotler, and Talluri) [36,37] with the estimated whole-body FFM in CT using the Mourtzakis equation [3]:

- Kanellakis-estimated FFM [36] had a strong linear relation with Mourtzakis [3] (*r* = 0.836, *p* < 2.2 × 10^−16^) (Figure 7A). Quantitative agreement was poor (*ρ* = 0.468, 95%CI: 0.396, 0.534). Kanellakis underestimated FFM with a 12.5 (6.7) kg systematic bias, a linear regression model compatible with a dose-dependent bias, (−0.6, 2.6) kg LoA, and 9/156 (5.7%) measurements outside the LoA (Figure 7B). Grouped measurements were significantly different (32.6 vs. 42.4 kg, *p* < 2.2 × 10^−16^) (Figure 7C).
- Kotler-estimated FFM [37] had a strong linear relation with Mourtzakis [3] (*r* = 0.802, *p* < 2.2 × 10^−16^) (Figure 7D). Quantitative agreement was poor (*ρ* = 0.718, 95%CI: 0.643, 0.780). Kotler overestimated FFM with a −4.3 (5.7) kg systematic bias, no dose-dependent bias, (−15.4, 6.8) kg LoA, and 9/156 (5.7%) measurements outside the LoA (Figure 7E). Grouped measurements were significantly different (47.7 vs. 42.4 kg, *p* = 3.464 × 10^−16^) (Figure 7F).
- Talluri-estimated FFM had a strong linear relation with Mourtzakis [3] (*r* = 0.857, *p* < 2.2 × 10^−16^) (Figure 7G). Quantitative agreement was poor (*ρ* = 0.666, 95CI%: 0.595, 0.728). Talluri overestimated FFM with a −6.9 (5.0) kg systematic bias, no dose-dependent bias, (−16.7, 2.9) kg LoA, and 11/156 (7.0%) measurements outside the LoA (Figure 7H). Grouped measurements were significantly different (49.9 vs. 42.4 kg, *p* < 2.2 × 10^−16^) (Figure 7I).

#### 3.4.2. Agreement Between CT and US

We also tested if our dataset could replicate the USVALID (Fischer et al.) [38] equation using the same dependent and independent variables. In our dataset, the following equation with an adjusted R^2^ = 0.723 was developed (Table S3): L3-SMA(cm^2^) = −74.04 + 12.03 × Q + 0.56 × W + 0.70 × H + 18.86 × S, where Q is quadricipital muscle thickness using US (in cm), W is Weight (in kg), H is Height (in cm), and S is Sex (female = 0, male = 1).

**Figure 8:**
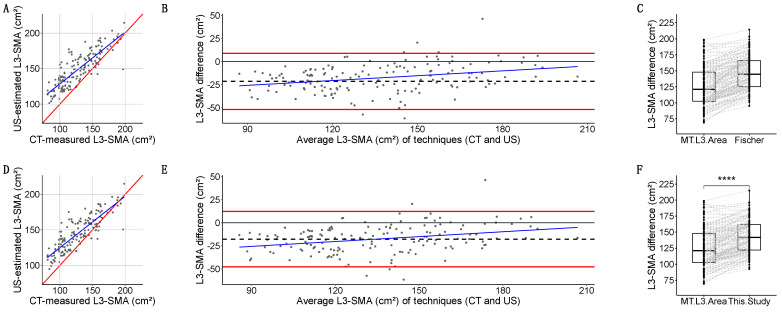
Scatterplot for CT-measured muscle CSA at L3 on the *X*-axis, and US-estimated muscle CSA at L3 on the *Y*-axis using the Fischer et al. equation [38] (**A**) and a similar regression equation with different ß coefficients (**D**). Bland–Altman plots for CT-measured muscle CSA at L3 on the X-axis, and US-estimated muscle CSA at L3 on the *Y*-axis using the Fischer et al. equation (**B**) and a similar regression equation with different ß coefficients (**E**). Boxplots with overlaid point geometry for CT-measured muscle CSA at L3 on the *X*-axis, and US-estimated muscle CSA at L3 on the *Y*-axis using the Fischer et al. equation (**C**) and a similar regression equation with different ß coefficients (**F**). Statistical significance for comparisons of central tendency measures is depicted as follows: *p* ≤ 0.0001 = ****.

Then, we compared the agreement of different equations for L3-SMA estimation using BIA (Fischer, and a new US-based regression model) with the measured L3-SMA in CT:

- Fischer-estimated L3-SMA [38] had a strong linear relation with L3-SMA (*r* = 0.849, *p* < 2.2 × 10^−16^) (Figure 8A). Quantitative agreement was poor (*ρ* = 0.642, 95%CI: 0.568, 0.705). Fischer overestimated L3-SMA with a −21.5 (15.2) cm^2^ bias, a dose-dependent bias in linear regression, and (−51.9, 8.9) kg LoA (Figure 8B). Grouped measurements were significantly different: 144.6 (40.7) vs. 121.2 (45.5) cm^2^ (*p* < 2.2 × 10^−16^) (Figure 8C).
- Our new equation had a strong linear relation with L3-SMA (*r* = 0.854, *p* < 2.2 × 10^−16^) (Figure 8D). Quantitative agreement was poor (*ρ* = 0.696, 95%CI: 0.626, 0.755). Our equation overestimated L3-SMA with a −17.6 (15.3) cm^2^ bias, a dose-dependent bias in linear regression, and (−47.6, 12.3) kg LoA (Figure 8E). Grouped measurements were significantly different: 141.7 (39.6) vs. 121.2 (45.5) cm^2^ (*p* < 2.2 × 10^−16^) (Figure 8F).

### 3.5. Impact of Techniques and Definitions on the Clinical Diagnosis of Muscle Atrophy

#### 3.5.1. Intra-Technique (Between Definitions) Agreement

In CT scans, muscle atrophy based on L3-SMA occurred in 10/156 (6.41%) cases and 76/156 (48.72%) cases applying the Van Vugt et al. [29] and Dolan et al. cut-off points [30], respectively. Qualitative agreement for muscle atrophy was slight, with *k* = 0.134 (*p* = 0.797 × 10^−3^). Using CT scans, it could be seen that there were 1/156 (0.64%) and 4/156 (2.56%) cases of sarcopenia applying the Van Vugt et al. [29] and Dolan et al. [30] cut-off points, respectively. Qualitative agreement for sarcopenia was fair, with *k* = 0.394 (*p* = 6.240 × 10^−10^). Myosteatosis based on L3-SMD occurred in 2/156 (1.28%) cases and 26/156 (16.66%) cases applying the Van Vugt et al. [29] and Dolan et al. [30] cut-off points, respectively. Qualitative agreement was slight, with *k* = 0.122 (*p* = 0.001). Although SMD was significantly lower in obese participants (120.8 vs. 140.3 HU, p = 0.001), there were no cases of myosteatosis using the Van Vugt et al. [29] definition in this group, with 2/116 cases of myosteatosis in the normal weight group. Using Dolan et al. [30] cut-off points, there were 10/40 (25.00%) cases of myosteatosis amongst obese participants, and 16/116 (13.79%) cases in the normal weight group.

Using Spanish (Masanés) [35] muscle atrophy cut-off points for BIA, muscle atrophy occurred in 38/156 cases (24.4%) and 3/156 cases (1.9%) using Janssen [34] or Talluri equations, respectively. Qualitative agreement between equations was non-existent, with *k* = −0.037 (n.s.) using Spanish (Masanés) [35] cut-off points. There were no cases of atrophy with neither equation using European [4,5] cut-off points. There was a total lack of qualitative agreement between muscle atrophy definitions, and therefore no qualitative agreement regarding sarcopenia.

In US, muscle atrophy based on RF-MT occurred in 16/156 cases (10.26%), while muscle atrophy based on RF-CSA took place in 13/156 cases (8.33%), with both cases using DRECO [28] cut-off points. Qualitative agreement was slight, with *k* = 0.127 (n.s.). Regarding sarcopenia, there were no cases with a RF-MT-based definition of muscle atrophy, and 2/156 (1.28%) cases with a RF-CSA-based based definition of muscle atrophy. Qualitative agreement for sarcopenia was non-existent in US.

#### 3.5.2. Inter-Technique Agreement

Muscle atrophy was defined in all CT cases using Van Vugt et al. [29] cut-off points. No agreement was found between CT and BIA in any case. When using Spanish (Masanés) [35] cut-off points, *k* = 0.07 (n.s.) for CT vs Janssen [34] equation, and *k* = −0.03 (n.s.) for CT vs Talluri equation. With European [4,5] cut-off points, agreement was non-existent with both BIA equations.

In general, CT and US displayed a slight agreement. When comparing CT muscle atrophy using Van Vugt et al. [29] cut-off points versus US muscle atrophy using DRECO cut-off points in RF-MT, a slight agreement was found, with *k* = 0.165 (*p* = 0.033). RF-CSA area also displayed a slight agreement with Van Vugt et al. [29] cut-off points (*k* = 0.109, n.s.). Results were slightly worse when comparing atrophy by Dolan et al. [30] according to L3-SMA measured on CT with the DRECO cut-off points: a slight agreement with RF-MT was found (*k* = 0.136, *p* = 0.005), yet there was no agreement with RF-CSA (*k* = 0.069, n.s.).

## 4. Discussion

Muscle atrophy and myosteatosis prevalence were generally low in a sample mainly composed of colorectal cancer survivors. The exception was the combinations of Janssen et al. [34] equation (BIA) with Masanés et al. [35] cut-off points, and L3-SMA and L3-SMD (CT) with Dolan et al. [30] cut-off points. Our inter-technique correlations were analogous to similar studies [28,51,52]. In our case, muscle atrophy and myosteatosis prevalence varied widely between techniques and definitions, as previously found [32,33,52]. This may be partly due to the intrinsic and different properties of each body composition technique. But more importantly, the current cut-off points for muscle atrophy and myosteatosis that have been used in the present study are intrinsically heterogeneous, since research groups and expert consensus have calculated them from different samples to act as: (1) a predictor of mortality, (2) a surrogate diagnosis for sarcopenia, or (3) a statistical deviation from a normal population. Therefore, we think that both the type of technique and its selected cut-off points should be taken into consideration when interpreting any body composition analysis study.

GLIM criteria [4,41] associated less muscle surface or mass in all techniques, thus proving a valid definition of malnutrition in our sample. Obesity did not associate worse muscle biomarkers per se. In fact, participants with this definition of obesity had significantly better muscle area or mass biomarkers in both CT and US. These results are in line with previous data that support the use of CT scans instead of a BMI-based approach to detect sarcopenic obesity in this study population [53]. Although some studies show the superiority of SMG as a muscle biomarker [54], we found no differences in SMG regarding the nutritional status of the participants in our sample. It is somehow striking that no statistically significant differences in mass or surface area between young and old participants were found, which may be explained by a modest age difference between groups (74.6 vs. 61.5 years) and sample size.

BIA and CT were not directly interchangeable in our study due to a BIA MM or FFM overestimation, as has happened in previous studies [32,33,52,55,56,57]. The Janssen et al. [34] equation presented the best diagnostic ability among the MM-estimating BIA equations in regards to whole-body MM estimation using the Shen [2] equation on L3-SMA, a finding also in line with previous evidence [32]. Nevertheless, the associated LoA were still excessively wide and Lin’s *ρ* was poor. The best FFM-estimating BIA equation in our sample was the Kotler et al. [37] equation, in comparison to a whole-body FFM estimation using the Mourtzakis et al. [3] equation on CT scans. With these exceptions, there was a generalized low agreement for muscle atrophy between CT and other valid and widespread BIA equations, both in quantitative and qualitative terms. This supports the recommendation that—if possible—population-specific BIA equations should be used [11,12], and their adequate functioning checked. Due to its intrinsic characteristics, foot-to-hand BIA overestimates the muscularity of the limbs and underestimates that of the torso [58]. This fact could partly explain the discrepancies between CT and BIA. In addition, some patients may be asymmetrically affected by “local sarcopenia” due to their underlying pathology and functionality [16]. In these subjects, body composition analysis techniques that compare skeletal muscle of the trunk (CT) versus that of the extremities (BIA and US) may not coincide, without this really being an error. Nevertheless, participants in this study had no comorbidities that may have caused “local sarcopenia”. We found no apparent bias due to the use of central catheters or ostomies, which may be frequent in the population of interest. Our correlation between SMD and raw phase angle was moderate and in the line of previous evidence in colorectal cancer [59].

US biomarkers levels were akin to those of another Spanish colorectal cancer study [51]. Analogous to the USVALID study [38], both CT and US adequately recognized a musculoskeletal sexual dimorphism in our work. As previously reported, GLIM malnutrition was associated with a lower RF-MT [60]. Despite its excellent methodological quality, the USVALID [38] equation displayed a suboptimal performance in our sample, even when muscle biomarkers were strikingly similar in both studies. These studies present differences in operators and measuring devices, and—more importantly—probe locations [61] that may not be interchangeable for skeletal muscle assessment [62]. Nevertheless, our US-based regression equation was equally suboptimal and unable to estimate L3-SMA using the same independent variables with adjusted ß coefficients for our study sample. Therefore, this lack of agreement between CT and US seems due to other reasons, such as patient morphotype. Taking all these findings this into consideration, CT and US were not directly interchangeable in our study, and further research on the validity and usefulness of US as a bedside body composition technique is warranted.

Our study has several strengths. To our knowledge, this is the first study that performed CT, BIA, US, and anthropometry simultaneously in colorectal cancer outpatients, as previous studies in this clinical population have worked with time spans up to one [33] or three months [51], or were conducted in a similarly very close period of time but in a different population [32,52,57]. On the other hand, all measurements were taken by a single trained operator (A.J.S.). This eliminates inter-observer bias, and also makes this a feasibility study, demonstrating that a well-trained endocrinologist could adequately perform all techniques. To our knowledge, this is also the first study that successfully uses the Butterfly iQ+^TM^ device (a handheld POCUS) to assess body composition in this clinical population. These devices may open new possibilities for quick, innocuous, and portable muscle assessment that may be of special interest when patients are unable to attend office appointments, such as hospitalized patients, or those in home care.

This study also has several limitations. Since this is a single-center study, the clinical and morphological characteristics of patients from other centers—and particularly from other countries—may not be superimposable on those of this study. Despite its careful methodology, this study was not pre-registered. Regarding malnutrition definition, our focus was quantitative and qualitative agreement between body composition analysis techniques (CT scans, BIA, and US) and not a traditional nutritional diagnosis, thus why a GLIM—instead of a Subjective Global Assessment (SGA)—approach was selected. Although it has some similarities with previous studies, this research is based on a slightly different population: instead of selecting referred patients to a Nutrition Unit before programed colorectal surgery [33,51], this study actively recruited colorectal cancer outpatients without taking into consideration neither their nutritional risk nor the natural history of the disease when they were invited to participate. Albeit we do not know whether the clinical and morphofunctional characteristics of the patients who did not wish to participate are different from those included, this blind inclusion of participants may have increased sample heterogeneity, potentially decreasing agreement but also increasing external validity. In this regard, patients with possible BIA artifacts (such as a central catheter, ostomy, and diuretic) were included in all analysis and regression equations. Therefore, we believe that these cases could not have favored one equation over the other. Although the inclusion of these participants may have increased our LoA in the Bland–Altman analysis, the artifact-free results were nearly superimposable on the whole sample in that regard, so it was not the case. Regarding US measurements, we do not know to what extent the use of a high-resolution device could have improved our results. Nevertheless, the DRECO study also used a handheld POC US (UProbe L6C) [28]. Besides, differences of vastus lateralis muscle thickness using a POC US vs. a gold-standard device were very small, with substantial or near-perfect intra-rater agreement in previous research [63].

In our study, the diagnosis of muscular atrophy using current definitions was not sufficiently congruent within and between techniques to justify the interchangeable use of these definitions in a similar population. More importantly, quantitative agreement between reference and bedside techniques was not sufficiently intense, accurate, and unbiased to justify the interchangeable use of these techniques in these patients in clinical practice. If a reference technique (CT) is not available, we therefore suggest using a single bedside technique, with a single raw parameter or equation, and a single set of cut-off points, for a more homogeneous follow-up of the muscular status of these patients. We consider that this technique should be chosen based on availability, operator experience, and possible artifacts or contraindications in the individual patient: for example, US could lose definition in patients with an abnormally large subcutaneous adipose panniculus, or BIA could be unreliable in patients with volume overload. As a future line of research, we believe that these limitations could be overcome or mitigated with prospective multicenter studies where both reference and bedside techniques are simultaneously performed, stratifying data by race, gender, and disease status, amongst others. This approach would allow for adjusted prognostic cut-off points on the pure measurements obtained with each technique; as well as regression equations that could transform the results of one technique into another with the highest possible precision, although the use of prediction equations will always introduce a new layer of uncertainty. If the latter were not significantly better than the currently available equations, then a second-best approach could be the stratification of data to find groups of patients where equation performance was sufficiently accurate and unbiased for a direct clinical application.

## 5. Conclusions

In general, intra-technique agreement with usual operationalized definitions of muscular atrophy was low. Inter-technique agreement regarding the diagnosis of muscular atrophy was equally low. Neither CT and BIA, nor CT and US, were interchangeable. Nevertheless, all techniques adequately recognized the same body composition patterns in the study sample, thus demonstrating biologic plausibility. It seems necessary to continue developing new equations and homogenizing cut-off points with multicenter, prognostic, and prospective studies that may improve agreement between body composition analysis techniques in colorectal cancer outpatients.

## Figures and Tables

**Table 1:** Muscle biomarkers, definitions of muscle atrophy, and myosteatosis.

Technique	Muscle Biomarker	European Cut-Off Points
CT	SMI-CT (cm^2^/m^2^) = L3-SMA/H^2^	Van Vugt et al. [29]: See Table A1 Dolan et al. [30]: <45 cm^2^/m^2^ if BMI < 25 kg/m^2^ ♂, <53 cm^2^/m^2^ if BMI ≥ 25 kg/m^2^ ♂, <39 cm^2^/m^2^ if BMI < 25 kg/m^2^ ♀, <41 cm^2^/m^2^ if BMI ≥ 25 kg/m^2^ ♀
	SMD (HU)	Van Vugt et al. [29]: See Table A1 Dolan et al. [30]: <34 HU if BMI < 25.0 kg/m^2^ in both sexes, <32 HU if BMI ≥ 25 kg/m^2^ in both sexes
	SMG (AU) = SMI-CT × SMD [31]	NA
	Mourtzakis et al. [3]; FFM (kg) = 0.30 × L3-SMA + 6.06	
	Shen et al. MM [2,32,33]; MM (kg) = [(0.166 × L3-SMA + 2.142)] × 1.06; SMI-Shen (kg/m^2^) = MT-mass/H^2^	
BIA	Janssen et al. [34]; MM (kg) = 5.102 + [0.401 × (H^2^/R)] + (3.825 × S) − (0.071 × A); SMI-Janssen (kg/m^2^) = SMM-Janssen/H^2^	Masanés et al. [35] (Spanish): <8.31 Kg/m^2^ ♂, <6.68 Kg/m^2^ ♀ European [4,5]: <7.00 Kg/m^2^ ♂, <5.50 Kg/m^2^ ♀
	MM-Talluri (kg) ^c^	NA
	Kanellakis et al. [36]; FFM (kg) ^a^ = 12.299 + 0.164 × W + 7.287 × S − 0.116 × (R_z_/H) + 0.365 × (X_c_/H^2^) + 21.570 x H	
	Kotler et al. [37]; FFM (kg) ^b^ = 0.88 × [(H^2.24^/Z^0.63^) × (1.0/37.63)] + 0.16 × W − 3.96	
	FFM-Talluri (kg) ^c^	
US	RF-MT (mm)	DRECO study, de Luis et al. [28]: <9.66 mm ♂, <10.4 mm ♀
	RF-CSA (cm^2^)	DRECO study, de Luis et al. [28]: <3.48 cm^2^ ♂, <2.4 cm^2^ ♀
	Quad-MT (mm)	NA
	Fischer et al. (USVALID) [38]; L3-SMA (cm^2^) ^d^ = −54.0 + (21.0 × S) + (0.4 × W) + (0.6 × H) + (15.0 × Quad-MT)	NA

♀: female; ♂ male; A: age in years; AU: arbitrary units; BIA: bioelectrical impedance; BMI: Body Mass Index; cm: centimeters; FFM: fat-free mass, H: height; HU: Hounsfield units; kg: kilograms; mm: millimeters; L3-SMA: skeletal muscle area at L3, L3-SMD: skeletal muscle density at L3, NA: not available; MM: muscle mass; RF-CSA: rectus femoris cross-sectional area in mm; RF-MT rectus femoris muscle thickness in mm; R_z_: resistance at 50 kHz in ohm (Ω); S: sex (female = 0, male = 1); SMI: Skeletal Muscle Index; US: nutritional ultrasound^®^; W: weight in kg; X_c_: reactance at 50 kHz (Ω); Z: impedance at 50 kHz in ohm (Ω). ^a^ H expressed in m; ^b^ H expressed in cm; ^c^ Proprietary equations are based to a significant degree on computed algorithms developed by Sun S. et al. [39]; ^d^ H expressed in cm and Quad-MT expressed in cm.

**Table 2:** Clinical and demographic characteristics of the study sample.

Parameter	Results
Female	*n* = 75 (48.1%)
Age (years)	M = 65.2 (13.6)
70 years or older	*n* = 54 (34.6%)
Height (m)	$\bar{x}$ = 1.64 (0.09)
Weight (kg)	M = 73.3 (19.9)
BMI (kg/m^2^)	M = 27.3 (5.7)
Obesity	Yes, *n* = 41 (26.3%) No, *n* = 115 (73.7%)
BMI as factor	Underweight, *n* = 8 (5.1%) Normal weight, *n* = 50 (32.0%) Overweight, *n* = 57 (36.5%) Grade 1 obesity, *n* = 27 (17.3%) Grade 2 obesity, *n* = 10 (6.4%) Grade 3 obesity, *n* = 4 (2.6%)
GLIM malnutrition	Yes, *n* = 11 (7.0%) No, *n* = 145 (93.0%)

*n_i_*: absolute frequency; %: percentage; kg: kilograms; $\bar{x}$ (): mean (standard deviation); M (): median (interquartile range); m: meters.

**Table 3:** CT-based muscle biomarkers.

	L3 SMA (cm^2^)	L3 SMD (HU)	L3 SMI (cm^2^/m^2^)	SMG (AU)	Shen MM (kg) [2]	Mourtzakis FFM (kg) [3]
Whole sample	$\bar{x}$: 125.8 (29.4)M: 121.2 (45.5) [69.2, 198.6]	$\bar{x}$: 40.7 (8.2) M: 40.8 (10.6) [21.8, 62.8]	$\bar{x}$: 46.3 (8.6) M: 44.9 (12.7) [28.5, 71.3]	$\bar{x}$: 1889.5 (520.6) M: 1873.6 (744.9) [768.3, 3374.8]	$\bar{x}$: 24.4 (5.2) M: 23.6 (8.0) [14.4, 37.2]	$\bar{x}$: 43.8 (8.8) M: 42.4 (13.7) [26.8, 65.6]
Sex Male Female *p*	145.2 103.4 <2.2 × 10^−16^	40.3 41.1 0.576	51.4 41.0 1.129 × 10^−11^	2052.4 1713.6 2.886 × 10^−5^	27.8 20.50 <2.2 × 10^−16^	49.6 37.1 <2.2 × 10^−16^
Age Young Elder *p*	121.2 120.2 0.317	42.7 36.9 2.034 × 10^−5^	44.7 45.2 0.969	1988.2 1703.2 7.482 × 10^−4^	23.6 23.4 0.317	42.1 42.4 0.317
Obesity No Yes *p*	117.8 146.0 0.001	120.5 140.7 8.243 × 10^−3^	43.5 51.7 3.833 × 10^−7^	1862.5 1965.4 0.288	23.0 28.0 0.001	41.4 49.9 0.001
GLIM Negative Positive *p*	122.9 108.4 0.041	40.2 47.0 0.059	45.0 39.3 0.005	1892.3 1853.8 0.804	23.9 21.3 0.041	42.9 38.6 0.041

The *p*-value (*p*) for the associated central tendency tests in each case is displayed on the right of each category (*t*-test if assumptions were met, Wilcoxon signed-rank test if not). AU: arbitrary units; cm: centimeters; FFM: fat-free mass; GLIM: Global Leadership Initiative in Malnutrition; HU: Hounsfield units; MM: muscle mass; *p*: *p*-value. Range is represented as [minimum, maximum].

**Table 4:** BIA muscle biomarkers.

	**Janssen MM (kg)** [34]	**Talluri MM (kg)**	**Kanellakis FFM (kg)** [36]	**Kotler FFM (kg)** [37]	**Talluri FFM (kg)**	**θ (°)**
Whole sample	$\bar{x}$: 22.9 (6.1) M: 23.3 (9.2) [13.6, 37.1]	$\bar{x}$: 30.8 (7.0) M: 29.9 (9.4) [15.2, 50.5]	$\bar{x}$: 31.3 (12.0) M: 32.6 (19.1) [6.4, 58.2]	$\bar{x}$: 48.1 (9.2) M: 47.7 (13.1) [30.6, 74.5]	$\bar{x}$: 50.7 (9.7) M: 49.9 (14.2) [33.4, 80.1]	$\bar{x}$: 5.0 (0.7) M: 5.1 (0.9) [2.6, 6.7]
Sex Male Female * p*	26.7 17.5 <2.2 × 10^−16^	34.9 26.0 <2.2 × 10^−16^	40.3 21.1 <2.2 × 10^−16^	52.8 40.7 <2.2 × 10^−16^	56.8 42.6 <2.2 × 10^−16^	5.3 4.9 0.002
Age Young Elder *p*	23.5 22.3 0.287	29.9 29.0 0.103	33.5 32.0 0.865	47.7 47.9 0.719	50.4 49.1 0.807	5.3 4.7 2.44 × 10^−6^
Obesity No obesity Obesity *p*	22.5 26.1 0.041	29.4 33.5 8.258 × 10^−3^	30.5 38.2 1.596 × 10^−3^	45.7 53.0 7.969 × 10^−4^	48.5 55.2 5.229 × 10^−4^	5.2 4.8 0.277
GLIM Negative Positive *p*	23.5 18.5 0.050	30.6 23.5 5.171 × 10^−4^	33.5 18.8 0.003	48.7 41.5 0.006	50.5 41.4 9.361 × 10^−4^	5.1 4.90.327

°: degrees; FFM: fat-free mass; θ: raw phase angle.

**Table 5:** US-based muscle biomarkers.

	RF-MT (mm)	Quad-MT (mm)	RF-CSA (cm^2^)	HGS (kg)
Whole sample	$\bar{x}$: 14.3(3.3) M: 13.9(3.9) [7.1, 25.9]	$\bar{x}$: 27.3(7.0) M: 26.6(9.1) [11.7, 48.9]	$\bar{x}$: 4.2(1.2) M: 4.1(1.4) [1.7, 8.3]	$\bar{x}$: 34.8 (10.0) M: 33.9 (12.9) [13.3, 64.0]
Sex Male Female *p*	15.4 12.5 5.154 × 10^−9^	29.9 23.8 6.309 × 10^−9^	4.64 3.60 8.208 × 10^−9^	40.0 29.0 1.7 × 10^−13^
Age Young Elder *p*	14.4 13.5 0.082	26.7 25.1 0.214	4.19 3.79 0.121	36.4 31.6 0.006
Obesity No obesity Obesity *p*	13.3 14.6 3.164 × 10^−3^	25.1 30.0 8.568 × 10^−3^	3.9 4.5 1.794 × 10^−3^	34.6 35.5 0.655
GLIM Negative Positive *p*	14.4 11.7 7.204 × 10^−3^	27.1 22.1 2.502 × 10^−4^	4.18 3.26 0.002	35.0 32.1 0.407

HGS: maximal handgrip strength; RF-CSA: rectus femoris Cross-Sectional Area; RF-MT: rectus femoris muscle thickness; Quad-MT: quadricipital (rectus femoris plus vastus intermedius) muscle thickness.

## Data Availability

The data presented in this study are available on request from the corresponding author as per European legislation on data protection.

## Supplementary Materials

The following supporting information can be downloaded at: https://www.mdpi.com/article/10.3390/nu16244312/s1, Figure S1: Density plots for body mass index (BMI); Figure S2: Bland–Altman plots stratified by possible BIA artifacts. Table S1: Possible BIA artifacts; Table S2. Tumor-related characteristics; Table S3. US-based regression model for L3-SMA.

## Appendix A

**Table A1:** Van Vugt et al. cutoff points [29] for muscle atrophy and myosteatosis in a European population.

	Men				Women			
	BMI (kg/m^2^)				BMI (kg/m^2^)			
	Skeletal muscle index (cm^2^/m^2^)							
Age (years)	17–20	20–25	25–30	30–35	17–20	20–25	25–30	30–35
30–39	36.8	41.3	47.0	52.6	31.0	33.9	37.4	40.9
40–49	35.4	39.9	45.6	51.2	31.0	33.5	36.5	39.5
50–59	34.0	38.6	44.2	49.8	31.0	33.0	35.5	38.0
60–69	32.6	37.2	42.8	48.4	30.9	32.5	34.5	36.4
70–79	31.2	35.8	41.4	47.0	30.7	32.0	33.5	34.8
80–87	29.8	34.4	40.0	45.6	30.6	31.5	32.5	33.2
	Skeletal muscle density (HU)							
30–39	42.5	39.8	36.3	32.8	41.0	37.7	33.6	29.4
40–49	39.6	36.8	33.4	29.9	37.2	34.0	29.8	25.6
50–59	36.6	33.9	30.5	27.0	33.4	30.1	26.0	21.8
60–69	33.7	31.0	27.5	24.0	29.6	26.3	22.2	18.0
70–79	30.7	28.0	24.5	21.1	25.8	22.5	18.3	14.1
80–87	27.7	25.0	21.6	18.1	21.9	18.6	14.4	10.3

Modified from Van Vugt et al. [29]. BMI: Body Mass Index; cm: centimeters; HU: Hounsfield units; kg: kilograms; m: meters.
