# Supplementary material for: Muscle Biomarkers in Colorectal Cancer Outpatients: Agreement Between Computed Tomography, Bioelectrical Impedance Analysis, and Nutritional Ultrasound
Source: Nutrients. 2024 Dec 13;16(24):4312. doi: 10.3390/nu16244312 (PMC11677386; doi:10.3390/nu16244312)
Supplement: Supplementary file 1 [file nutrients-16-04312-s001.zip › nutrients-3348669-supplementary.pdf]

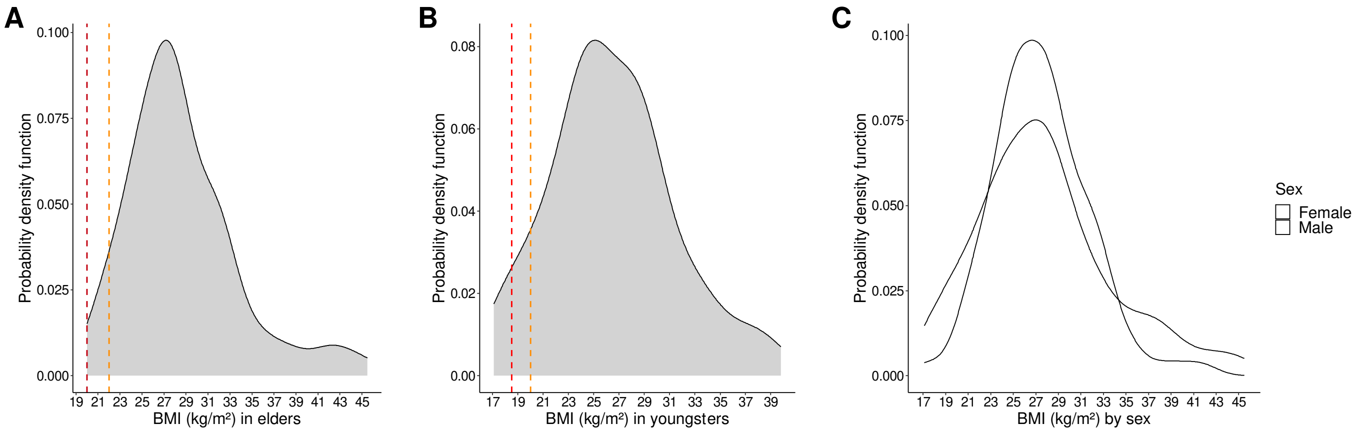

Figure S1. Density plots for body mass index (BMI). Data is stratified by age: < 65 years (A); > 65 years (B); and by sex (C). A vertical, dashed, and red line represents in (A) and (B) the correspondent BMI for "severe malnutrition" in GLIM criteria. A vertical, dashed, and orange line represents in (A) and (B) the correspondent BMI for "moderate malnutrition" in GLIM criteria. GLIM: Global Leadership Initiative on Malnutrition.

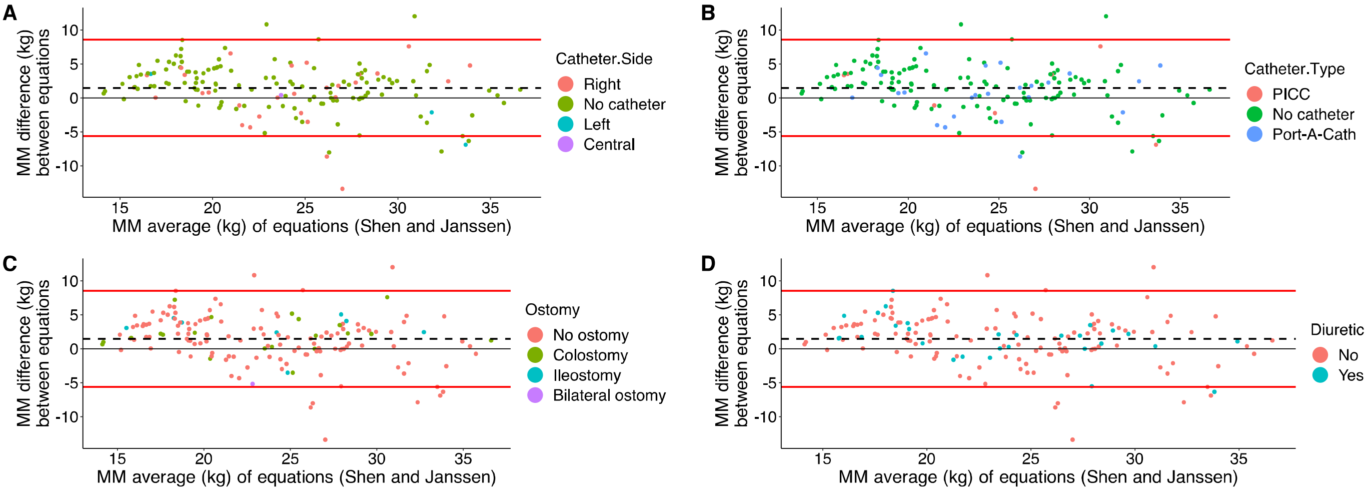

Figure S2. Bland-Altman plots for CT-based MM using the Shen equation, in comparison with BIA-based MM using the Janssen equation. Data has been colored depending of the presence of different possible artiphacts present at physical exploration: presence of central catheter (A), type of central catheter (B), presence of ostomy (C), and presence of chronic treatment with a diuretic drug (D). CT: computed tomography; kg: kilogram; MM: muscle mass; PICC: Peripherically Inserted Central Catheter.

Table S1. Possible BIA artifacts

Parameter	Results
Central catheter	No catheter, *n* = 123 (78.8%)<br>PICC, *n* = 8 (5.1%)<br>Port-A-Cath, n = 25 (16.0%)
Central catheter side	Central, n = 1 (0.6%)<br>Left, *n* = 3 (1.9%)<br>No catheter, *n* = 123 (78.8%)<br>Right, *n* = 29 (18.6%)
Ostomy	Bilateral ostomy, *n* = 1 (0.6%)<br>Colostomy, *n* = 21 (13.5%)<br>Ileostomy, *n* = 8 (5.1%)<br>No ostomy, *n* = 126 (80.8%)
Metallic prothesis	Bilateral, *n* = 2 (1.3%)<br>Contralateral, *n* = 8 (5.1%)<br>No prothesis, *n* = 146 (93.6%)
Diuretic	No, *n* = 126 (80.7%)<br>Yes, n = 30 (19.2%)

ECOG: Eastern Cooperative Oncology Group performance status

Table S2. Tumor-related characteristics

Parameter	Results
ECOG	0, *n* = 101 (64.7%)<br>1, *n* = 50 (32.0%)<br>2, *n* = 4 (2.6%)<br>3, *n* = 1 (0.7%)
Neoplasm location	Right colon, *n* = 34 (21.8%)<br>Transverse colon, *n* = 12 (7.7%)<br>Left colon, *n* = 19 (12.2%)<br>Rectosigmoid, *n* = 13 (8.3%)<br>Sigma, *n* = 32 (20.5%)<br>Rectum, *n* = 45 (28.8%)<br>Krukenberg, *n* = 1 (0.7%)
Stage (TNM) at diagnosis	IIA (*n* = 27); IIB (*n* = 3); IIC (*n* = 4)<br>IIIA (*n* = 12); IIIB (*n* = 48); IIIC (*n* = 16)<br>IVA (*n* = 20); IVB (*n* = 15); IVC (*n* = 11)
Previous surgery	Yes, *n* = 143 (91.7%)<br>No, *n* = 13 (8.3%)
First surgery	Anexectomy, *n* = 1 (0.7%)<br>Abdomino-perineal resection, *n* = 8 (5.6%)<br>Colostomy, *n* = 5 (3.5%)<br>Exploratory laparotomy, *n* = 1 (0.7%)<br>Fistulotomy, *n* = 1 (0.7%)<br>Hepatectomy, *n* = 6 (4.2%)<br>High anterior resection, *n* = 1 (0.7%)<br>Left hemi-colectomy, *n* = 17 (11.9%)<br>Low anterior resection, *n* = 34 (23.8%)

Table S2. *Cont.*

Parameter	Results
First surgery	Right hemi-colectomy, *n* = 37 (25.9%)<br>Sigmoidectomy, *n* = 26 (18.1%)<br>Sub-total colectomy, *n* = 6 (4.2%)
Active chemotherapy	Yes, *n* = 38 (24.4%)<br>No, *n* = 118 (75.6%)
Chemotherapy	Neoadjuvant, *n* = 32 (20.5%)<br>Adjuvant, *n* = 98 (62.8%)<br>Initial treatment without surgery, *n* = 15 (9.6%)<br>No, *n* = 11 (7.0%)
First chemotherapy scheme	Capecitabine + bevacizumab, *n* = 1 (0.7%)<br>CAPOX, *n* = 2 (1.4%)<br>CAPOX + bevacizumab, *n* = 1 (0.7%)<br>CAPOX + capecitabine, *n* = 1 (0.7%)<br>Capecitabine, *n* = 49 (33.8%)<br>Cetuximab, *n* = 1 (0.7%)<br>FOLFOX, *n* = 10 (6.9%)<br>FOLFOX + bevacizumab, *n* = 9 (6.2%)<br>FOLFOX + cetuximab, *n* = 4 (2.7%)<br>FOLFOXIRI + bevacizumab, *n* = 2 (1.4%)<br>FUFA, *n* = 1 (0.7%)<br>XELOX, *n* = 60 (41.4%)<br>XELOX + bevacizumab, *n* = 4 (2.7%)
Radiotherapy	Neoadjuvant, *n* = 29 (18.6%)<br>Adjuvant, *n* = 18 (11.5%)<br>No, *n* = 109 (69.9%)

ECOG: Eastern Cooperative Oncology Group performance status

Table S3. US-based regression model for L3-SMA

Dependent: L3-SMA (cm^2)		Unit	Value	Coefficient (multivariate)
Sex	Female (N=75)	Mean ± SD	105.0 ± 19.6	
	Male (N=81)	Mean ± SD	145.1 ± 23.2	18.86 (12.10 to 25.61, $p < 0.001$)
Weight (kg)	[39.2,133.1]	Mean ± SD	74.5 ± 15.2	0.56 (0.35 to 0.76, $p < 0.001$)
Height (cm)	[146.4,183.7]	Mean ± SD	164.2 ± 8.9	0.70 (0.33 to 1.08, $p < 0.001$)
Quadricipital muscle thickness (cm)	[1.2,4.9]	Mean ± SD	2.7 ± 0.7	12.03 (7.61 to 16.45, $p < 0.001$)

Multivariate linear regression with CT-based L3-SMA as dependent variable and sex, weight, height and quadricipital muscle thickness (*rectus femoris* plus *vastus intermedius*) in the whole sample, using the same variables as the USVALID study [49]. Adjusted $R^2 = 0.723$, $p < 2.2 \times 10^{-16}$. Please not how we have used the same units as Fischer et al. for an easier comparison.
